# Determination of azithromycin heteroresistant *Campylobacter jejuni* in traveler’s diarrhea

**DOI:** 10.1186/s13099-019-0301-1

**Published:** 2019-05-06

**Authors:** Woradee Lurchachaiwong, Supaporn Ruksasiri, Patcharawalai Wassanarungroj, Oralak Serichantalergs, Ladaporn Bodhidatta, John Crawford, Sanjaya Kumar Shrestha, Prativa Pandey

**Affiliations:** 1 Department of Bacterial and Parasitic Diseases, Armed Forces Research Institute of Medical Sciences, 315/6 Rajvithi Road, Bangkok, 10400 Thailand; 2 Walter Reed AFRIMS Research Unit, Kathmandu, Nepal; 3 CIWEC Clinic Travel Medicine Center, Kathmandu, Nepal

**Keywords:** Heteroresistance, *Campylobacter jejuni*, Azithromycin, Travelers

## Abstract

*Campylobacter* is the most common cause of traveler’s diarrhea (TD) and human bacterial gastroenteritis. A heteroresistant *Campylobacter jejuni* (*C. jejuni*) isolate, identified by microbiological methods and characterized with molecular techniques, was obtained from a traveler in Nepal suffering TD. The presence of atypical colonies within the clear zone of inhibition was the first evidence of an atypical phenotype, leading to additional characterization of this heteroresistant strain. Antimicrobial susceptibility testing (AST) and population analysis profiling (PAP) demonstrated heteroresistance to azithromycin (AZM), a first-line antibiotic treatment for *Campylobacter* infections. Molecular analysis indicated a point mutation occurred on the 23S rRNA gene at the A2075G transitions, and the number of mutated gene copies was proportional to AZM resistance. Heteroresistant *C. jejuni* subpopulations from acute TD are likely underestimated, which may lead to treatment failures, as was the case for this patient. The presence of a heteroresistant strain in a high antibiotic environment may select for additional drug resistance and enable distribution into hospital and local communities.

## Background

*Campylobacter* is a Gram-negative bacteria causing travelers’ diarrhea (TD) in US military personnel during deployment to developing countries [1], as well as a leading cause of TD in Nepal and bacterial foodborne illness worldwide [2, 3]. The vast majority of these intestinal infections are caused by *Campylobacter jejuni* (*C. jejuni*) and *Campylobacter coli* (*C. coli*), of which approximately 90% are attributed to *C. jejuni* [3]. Macrolides, primarily azithromycin (AZM) or erythromycin (ERY), remain the frontline agents for treating *Campylobacter* infections [4]; however, a progressive increase in *Campylobacter* macrolide resistance was reported in some countries and is a growing health threat concern [5]. The term “heteroresistance” describes a phenomenon where subpopulations of seemingly isogenic bacteria exhibit a range of susceptibilities to a particular antibiotic [6]. The mechanism of action with macrolides resistance was traced to a mutation on domain V of the 23S rRNA. Single base substitution positions, as well as the number of mutated 23S rRNA copies, are correlated to macrolide resistance and proportion levels of minimum inhibitory concentrations (MICs) [7]. As a result of genotypic variation, the Clinical and Laboratory Standards Institute (CLSI) developed clinical laboratory standards and recommendations regarding antimicrobial resistance [6]. Until recently, heteroresistant patterns were poorly understood and the application of consensus-based standards for this phenotype was controversial. Confusion on this phenomenon precludes establishing its clinical significance, leading to improper therapeutic interventions and ambiguous guidelines. Not recognizing AZM heteroresistance in *C. jejuni* may lead to treatment failure, and as such lead to increasing resistance. Hence, this study characterized AZM heteroresistant *C. jejuni* from acute diarrheal illness in travelers to Nepal. The early detection of heteroresistant *Campylobacter* isolates enables medical providers to make appropriate treatment decisions.

## Methods

A total of 105 *Campylobacter* isolates, including 88 *C. jejuni* and 17 *C. coli* were obtained from 105 travelers with acute diarrheal illness seeking healthcare at the CIWEC Clinic Travel Medicine Center in Kathmandu, Nepal during 2012–2014. Antimicrobial susceptibility testing (AST) to azithromycin (AZM), erythromycin (ERY), nalidixic acid (NAL), and ciprofloxacin (CIP) was performed on confirmed isolates using a commercially available E-test method (Liofilchem, Roseto degli Abruzzi TE, Italy) to determine the minimal inhibitory concentration (MIC). The AST results were interpreted following CLSI guidelines and National Antimicrobial Resistance Monitoring System (NARMS) using *Campylobacter jejuni* ATCC 33560 as the control strain [8, 9]. AST observations of the heterogeneous phenotype was the presence of discrete colonies in the clear zone of inhibition. These isolates were recorded as “suspected heteroresistance” and the discrete colonies were harvested for heteroresistant determinations. The colonies showing suspected heteroresistance were submitted for capsule genotyping by multiplex PCR [10]. The colonies from the same specimens that have the same capsule genotypes, but discriminate AST profiles, were subsequently assessed with the “population analysis profiling (PAP)” which remains the gold standard for detecting heteroresistance. Isolates were determined to be heteroresistant when the lowest antibiotic concentration giving the maximum growth inhibition was eightfold higher than the highest non-inhibitory concentration [6, 11, 12]. Furthermore, these isolates were sequenced at the domain V of the 23S rRNA gene, as described elsewhere [7]. Briefly, genomic DNA extraction for *C. jejuni* and *C. coli* was be prepared using the DNeasy Blood & Tissue kit (Qiagen Valencia, CA) according to manufacturer’s directions. Primers flanking each operon were utilized to amplify the three copies of the 23S rRNA gene in *C. coli* and *C. jejuni* isolates. Amplified products were analyzed by gel electrophoresis and ethidium bromide staining was used to determine the amplicon sizes. The amplified products were further purified using the Wizard PCR purification system (Promega, Madison, WI) and submitted for sequencing using primers specific for the 23S domain V. The 508 bp of 23S rRNA sequences were analyzed to identify potential macrolide mutations in each copy target gene by Sequencher version 5.0 DNA sequence analysis software (Gene Codes Corporation, Ann Arbor, MI).

## Results and discussion

Of 105 *Campylobacter* isolates, one *C. jejuni* was found to be heteroresistant to AZM. This acute diarrheal stool sample came from a traveler who presented to the clinic with fever, abdominal pain, nausea, vomiting, diarrhea and fatigue. The stool characteristic was watery without mucus and blood. The sample was negative for enteric viruses, gastrointestinal parasites, and enteric bacterial pathogens including diarrheagenic *E. coli*; however, colonies identified as *C. jejuni* were detected.

**Fig. 1:**
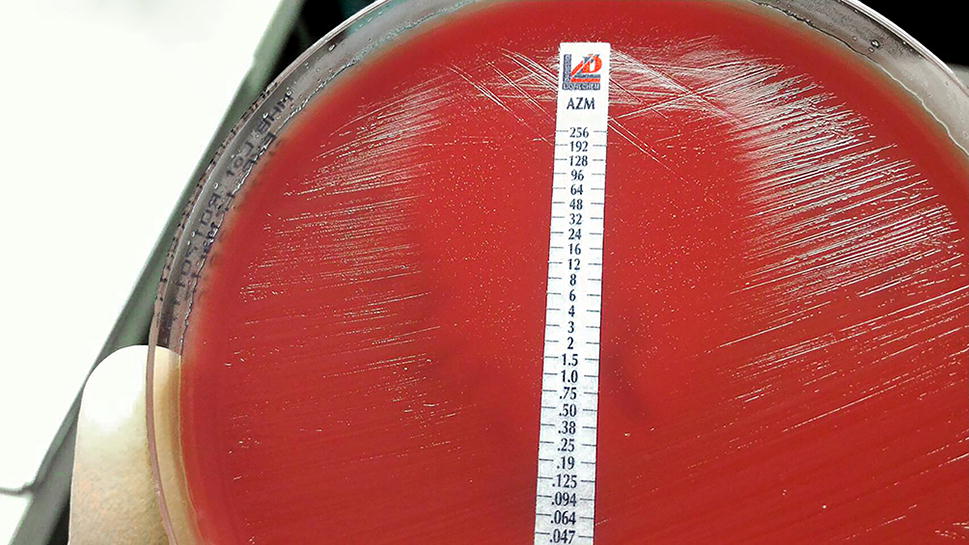
The antimicrobial susceptibility result of heteroresistant *C. jejuni* to AZM, presenting as a distribution of speckle colonies within the inhibition zone

The AST profile of this heterogeneous *C. jejuni* isolate was MICs ≥ 256 μg/ml to CIP, ERY, and NAL, and presenting resistance to CIP, ERY, and NAL, respectively. This *Campylobacter* isolate displayed a heterogeneous phenotype by presenting as speckle colonies in the clear zone of inhibition to AZM. Repetitive AST testing to AZM of the original *Campylobacter* isolate, as well as colonies from the outer and the inner (speckle colonies) inhibition zone, were confirmed by E-test to make the heteroresistant determination. The results were reproducible, presenting with a MIC = 0.19 μg/ml; however, the speckle colonies on the inner inhibition zone showed inconsistent MIC result to AZM (Fig. 1). Subsequently, the original *Campylobacter* isolate, colonies from outer inhibition zone, and three random speckle colonies from the inner inhibition zone were submitted for capsule genotyping by multiplex PCR. All isolates possessed the same capsule type to HS2, which potentially has a common Penner type of *C. jejuni* isolated from South and Southeast Asia [10], as well as to a previous report of fluoroquinolone resistance in Hawaii [13].

**Fig. 2:**
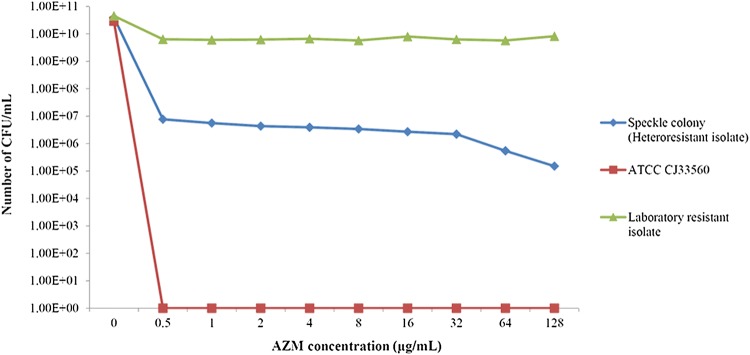
The population analysis profiling (PAP) result of the laboratory resistant isolate, heteroresistant isolate, and ATCC 33560 control strain performed at the different AZM concentrations from 0 to 128 µg/ml by measuring the number of colony forming units (CFU)

Regarding the PAP result, the laboratory resistant and heteroresistant isolates exhibited different growth patterns between the lowest concentrations to maximum inhibition and the highest non-inhibitory concentration when compare to the ATCC 33560 *C. jejuni* control strain (Fig. 2). The laboratory isolate demonstrated persistent resistance to AZM, whereas the heteroresistant isolate gradually reduced the number of bacteria from 10^11^ CFU to 10^5^ CFU when the AZM concentration was increased to 128 µg/ml. This isolate was suspected as heteroresistant due to a more than eightfold difference between the lowest concentrations exhibiting maximum inhibition and the highest non-inhibitory concentration. Additionally, the AZM heterogeneous *C. jejuni* isolate genotype was compared with resistant *C. jejuni*, resistant *C. coli*, and an ATCC control strain (Table 1). At least one mutation was detected in all of the macrolide-resistant strains, but no mutation was observed in the ATCC 33560 control. Heterogeneous *C. jejuni* and resistant *C. jejuni* isolates possessed point mutations of 23S rRNA genes at positions 2075, whereas resistant *C. coli* presented the mutation at position 2230. The A2075G transitions were previously reported as efflux systems [14], and the number of gene copy mutations were proportional to the increased level of macrolide resistance. The two copies of this mutated gene conferred a lower macrolide MIC than the *C. jejuni* isolate with mutations in all three copies, which was previously mentioned regarding its relationship to in vivo selective pressure [7].

**Table 1:** Determination of the minimal inhibitory concentration (MIC) and molecular characterization of the 23S rRNA gene mutations of heterogeneous *C. jejuni*, resistant *C. jejuni*, resistant *C. coli*, and *C. jejuni* ATCC 33560 against AZM

Organism	E-test to AZM	23S rRNA gene mutation at position 2075 (A2075G) for *C. jejuni*^b^			23S rRNA gene mutation at position 2230 (A2230G) for *C. coli*^c^		
		Copy I (FI)	Copy II (FII)	Copy III (FIII)	Copy I (FIa)	Copy II (FIIa)	Copy III (FIIIa)
*C. coli* ^c^	≥ 256 μg/ml				A2230G	A2230G	A2230G
*C. coli* ^c^	≥ 256 μg/ml				A2230G	A2230G	A2230G
*C. coli* ^c^	≥ 256 μg/ml				A2230G	A2230G	A2230G
*C. coli* ^c^	≥ 256 μg/ml				A2230G	A2230G	A2230G
*C. jejuni* ^b^	≥ 256 μg/ml	A2075G	A2075G	A2075G			
*C. jejuni* ^b^	≥ 256 μg/ml	A2075G	A2075G	A2075G			
*C. jejuni* ^b^	≥ 256 μg/ml	A2075G	A2075G	A2075G			
*C. jejuni* ^b^	≥ 256 μg/ml	A2075G	A2075G	A2075G			
*C. jejuni* (outer inhibition zone)^a^	0.19 μg/ml	No mutation	No mutation	A2075G			
*C. jejuni* (inner inhibition zone)^a^	≥ 256 μg/ml	A2075G	No mutation	A2075G			
ATCC33560	Susceptible	No mutation	No mutation	No mutation			

^a^Heterogeneous *C. jejuni*

^b^Resistant *C. jejuni* isolates a base substitution, G for A, were detected at position 2075 (corresponding to position 2059 in the nomenclature for *E. coli* [Genbank Accession No. J01695])

^c^Resistant *C. coli* isolates a base substitution, G for A, were detected at position 2230 (corresponding to position 2059 in the nomenclature or *E. coli* [Genbank Accession No. J01695], *C. coli* [Genbank Accession No. U09611])

This heteroresistant *C. jejuni* demonstrated high MICs to macrolides like AZM and ERY, quinolones like NAL, and fluoroquinolones like CIP. Macrolides, generally considered the drug of choice for *Campylobacter* treatment [1], may be clinically less effective for heteroresistant infections. It should be noted that patient of this acute diarrheal illness was treated with 500 mg of AZM for 3 days. Unfortunately, we were not able to obtain follow-up data regarding treatment results, so the effects of heteroresistance are not known. Nevertheless heteroresistant strains were previously reported that were responsible for treatment failure [15–18], which resulted in increased resistance to other antibiotics [19]. Due to the controversy of therapeutic implications of heteroresistance, relevant clinical data significance and additional experimental research is required [20]. Currently antimicrobial resistance in enteric pathogens is globally present. Despite this, the use of antimicrobial drugs in humans and animals are largely unrestricted [21]. The resistance to macrolides is more prevalent in *Campylobacter* isolates of animal origin, and the presence of macrolide-resistant isolates in the food chain increases the potential for human transmission [21]. Nonetheless, an increase of heteroresistant bacteria could be the result of high antibiotics use and possibly a driving mechanism for natural evolution to drug resistance [22] by proliferating and inhibiting susceptible subpopulations [20]. Hence, AST continues to play an important role in guiding therapy and epidemiological monitoring of resistance.

In conclusion, phenotypic and genotypic assessments of a *C. jejuni* isolate from a traveler to Nepal suffering acute diarrheal illness was confirmed as AZM heteroresistant. The conventional microbiology and phenotypic isolation techniques utilized for this study should be used to thoroughly examine atypical heteroresistant colonies. The original isolate, which presented as atypical colonies in the clear zone of inhibition, would suggest repetitive AST testing. The reproducible finding of atypical colonies in the clear zone of inhibition served as the primary observation of a heteroresistant strain, and the early detection of heteroresistant *Campylobacter* isolates enables medical providers to make appropriate treatment decisions. Regarding this knowledge gap, more research is needed to determine the true clinical impact of heteroresistance and its role in the development of fully-resistant bacterial populations. Proper laboratory and identification measures are needed to assess effective therapies and interventions for military members and civilian travelers suffering from persistent diarrhea.
